# Biodegradation of Pollutants in the Environment: Omics Approaches

**DOI:** 10.3390/ijms24108815

**Published:** 2023-05-16

**Authors:** Irina S. Moreira

**Affiliations:** CBQF—Centro de Biotecnologia e Química Fina, Laboratório Associado, Escola Superior de Biotecnologia, Universidade Católica Portuguesa, Rua Diogo Botelho 1327, 4169-005 Porto, Portugal; ismoreira@ucp.pt

This special edition intends to highlight how omics approaches have been used in biodegradation studies to understand the mechanisms involved and improve biodegradation processes. The use of genomics, tanscriptomics, proteomics, and metabolomics allows us to decipher the metabolic pathway and adaptations of bacteria in the presence of pollutants which allow them to tolerate and degrade the compounds. In the study of Wang et al. [1], the analysis of the transcriptome of *Xanthobacter* sp. YN2, along with the degradation of 1,4-dioxane (compared with citrate), identified glycoxylate as a key intermediate, which was further metabolized by three routes. The gene cluster for glyoxylate degradation was strongly upregulated by dioxane. Besides the expression of glycoxylase, the authors also reported the expression of methylotrophic genes. Moreover, genes related to quorum sensing and transporters were induced at the beginning of the process, while genes encoding two-component systems were significantly upregulated at the late stages of dioxane degradation. The high efficiency of this bacterium for 1,4-dioxane degradation seemed to be related to several factors, such as the constitutive expression of genes for degradative enzymes, resulting in the fast attack of the compound. Moreover, the existence of several routes and multiple enzymes for each step further accelerated the process.

In the study of Moreira et al. [2], the metabolic pathway of degradation for the endocrine disruptor 17-β estradiol by the bacterial strain *Rhodococcus* sp. ED55, in wastewater, was deciphered using metabolomics tools. The efficiency of *Rhodococcus* sp. ED55 in removing E2 in real wastewater was combined with the detection and identification of 17 metabolites, which was achieved by means of ultra-performance liquid chromatography coupled with high-resolution mass spectrometry (UPLC/ESI/HRMS). The time profile of the formed metabolites showed that the bioaugmentation with *Rhodococcus* sp. ED55 determined the faster and complete removal of produced intermediates. These results, combined with the elimination of toxicity and decrease in the estrogenic activity of the treated effluent, show the potential for the use of *Rhodococcus* sp. ED55 for environmental remediation processes.

The biodegradation of another endocrine disruptor, bisphenol A, by the fungus *Myrothecium roridum* IM 6482 was described by Jasińska et al. [3]. In this study, metabolomics (LC-MS/MS) was used to elucidate the metabolites produced during degradation, while proteomics (2-D electrophoresis and MALDI-TOF/TOF) were used to provide insights into the toxic effect of BPA on an intracellular level. It was verified that the efficient degradation of BPA, mainly through the activity of extracellular laccase, resulted in the production of hydroxylated derivatives, glucuronides, and dimmers, as transformation products, which presented lower estrogenic activity. In the presence of BPA, numerous proteins associated with the occurrence of oxidative stress, heat shock proteins, and oxidoreductases were detected. Oxidative stress was also verified by increased membrane permeability, the peroxidation of lipids, and the overproduction of reactive oxygen species.

Edwards et al. [4] identified the key genes implicated in polyethylene terephthalate (PET) synergistic degradation in a bacterial consortium of three *Pseudomonas* and two *Bacillus* spp. isolated from petroleum-contaminated soil. By employing pangenomic analysis, the authors identified not only the core gene clusters responsible for PET degradation but also other plastic- and plasticizer-degrading genes, suggesting the potential for the biodegradation of mixed plastic waste. RNA sequencing was used to identify the genetic pathways within the *Pseudomonas* and *Bacillus* spp. that, in part, explained the synergistic degradation of PET. An esterase encoded in the estB gene and identified within the pangenome was discovered. The new EstB PETase encoded in *Pseudomonas* spp. was observed to hydrolyze the oligomer BHET and the polymer PET.

The chronic toxic effect of the textile dyeing effluent (Monastir, Tunisia) on oxidative stress status and histological changes in male mice was evaluated in the study of Methneni et al. [5]. The tested effluent presented very high levels of the metals Cr, As, and Sr, and two textile dyes: a triphenylmethane dye (Crystal violet) and a disperse azo dye (Disperse yellow 3). After a 90-day exposure period, the generation of oxidative stress through the alteration of malondialdehyde, conjugated dienes, Sulfhydryl proteins, and catalase levels in the liver and kidney was observed, which may be attributed to the pathological lesions induced in these organs.

The separated and combined effects of metals and organic pollutants were also addressed in the study of Zaborowska et al. [6], who evaluated the impact of zinc and bisphenol A contamination on soil, namely on the microbiome. The results of this study revealed that a combined effect caused the strongest inhibitory impact on the enzymatic and microbiological activity and the diversity of microorganisms. The NGS analysis also exposed that some bacteria prevailed according to the type of contamination. Moreover, a negative effect was observed through the pollutants on the growth and development of *Sorghum Moench*, which was more affected than *Panicum virgatum* by soil contamination.

The potential of metatranscriptomic to help develop productive and sustainable agriculture was reviewed by Sharuddin et al. [7]. In this sense, it was reviewed how elucidating the functional role of the microbiome could be used to improve soil fertility and crop productivity, as well as the development of disease-suppressive soils as greener alternatives against biotic stress. The application of transcriptional profiles to monitor pollution via specific bioindicators and decrease negative impacts on the environment was also summarized.
